# Is There Limited Utility for Lifestyle Recommendations for Diabetes Prevention Among Overweight or Obese Depressed Patients?

**DOI:** 10.3389/fmed.2021.757250

**Published:** 2021-11-19

**Authors:** Arch G. Mainous, Benjamin J. Rooks, Frank A. Orlando

**Affiliations:** ^1^Department of Health Services Research, Management and Policy, University of Florida, Gainesville, FL, United States; ^2^Department of Community Health and Family Medicine, University of Florida, Gainesville, FL, United States

**Keywords:** diabetes risk, depression, NHANES, prevention, exercise

## Abstract

**Background:** Lifestyle interventions like diet and exercise are commonly recommended for diabetes prevention, but it is unclear if depression modifies the likelihood of adherence. We evaluated the relationship between high depressive symptomatology and adherence to lifestyle interventions among patients with pre-diabetes.

**Methods:** We conducted an analysis of the nationally representative National Health and Nutrition Examination Survey (NHANES) 2017–2018. Adults, aged ≥18 years old who were overweight or obese (BMI ≥25) and had diagnosed or undiagnosed pre-diabetes (HbA1c 5.7–6.4) were included. Depressive symptomatology was classified by the Patient Health Questionniare-9 (PHQ-9). We used self-reported adherence to physician suggested lifestyle changes of diet and exercise.

**Results:** In this nationally representative survey of overweight or obese adults with pre-diabetes, 14.8% also have high depressive symptomatology. In unadjusted analyses, an interaction was observed with high depressive symptomatology acting as an effect modifier for adherence to exercise oriented interventions among patients with diagnosed pre-diabetes (*p* = 0.027). In logistic regressions, adjusting for age, sex, race, outpatient medical care in the past 12 months, and obesity, among patients with diagnosed pre-diabetes, depressed patients were less likely to attempt to exercise more (OR = 0.31; 95% CI: 0.10, 0.94) and no association between high depressive symptomatology and attempting to lose weight was observed (OR = 0.45; 95% CI: 0.14, 1.42).

**Conclusions:** The findings of this nationally representative study of US adults, high depressive symptomatology decreases the likelihood of adherence to exercise based lifestyle recommendations among patients with diagnosed pre-diabetes.

## Introduction

Diabetes is a chronic, progressive disease that has reached epidemic proportions in the USA (1). The American Diabetes Association and United States Preventive Services Task Force recommend screening, early detection, and treatment for pre-diabetes as a necessary strategy to prevent diabetes and its severe complications (2–5). Diabetes prevention focuses on the use of lifestyle interventions and/or prescription metformin among the general population.

Individuals with depression are a vulnerable and important population in relation to diabetes and diabetes prevention. Diabetes and depression are common comorbid conditions and having both diabetes and depression is related to worse health outcomes than just having one of the conditions (6–8). Individuals with depression and pre-diabetes are at a greater risk of developing diabetes than those with only one of these risk factors (9–12).

Although lifestyle interventions are recommended and evidence-based for diabetes prevention, adherence to an intense lifestyle program may be difficult for depressed patients. Little research has focused on the adherence of lifestyle interventions for diabetes prevention among patients with pre-diabetes and depression (13). One of the few studies to investigate this question examined utilization of weight loss as strategy by patients but included people at normal weight, a population unlikely to try and increase exercise and try and lose weight. This is an important gap in our knowledge base because depressed patients are a particularly vulnerable group, and the standard approach for diabetes prevention, advice for lifestyle change, may not be an optimal strategy for depressed patients.

The purpose of this study was to investigate the adherence to physician reported diabetes prevention lifestyle interventions, increased exercise and weight loss, among overweight or obese adult patients with pre-diabetes and depression.

## Research Design And Methods

We analyzed the 2017–2018 National Health and Nutrition Examination Survey (NHANES). The NHANES is a large, nationally representative survey that samples the non-institutionalized population of the United States using a stratified multistage probability sample design. To account for nationally representative population estimates, the National Center for Health Statistics applies a multilevel weighting system. The survey included a standardized medical examination including blood analysis for examining biomarkers and a number of health-related interviews. The application of weights and variables accounting for the complex survey design allows the study to provide nationally representative population estimates for the United States. Our study focused on adults ≥18 years of age who were overweight or obese as defined by body mass index (BMI) of ≥25. BMI was obtained from body weight divided by height squared (kg/m^2^). Weight and height were measured by a trained examiner in the mobile examination center, and these were used to calculate BMI.

### Exclusions

We excluded patients who reported that they had undergone bariatric surgery since that would confound our assessment of lifestyle recommendations for weight loss. This was assessed by the individual's response to the question “Have you ever had weight loss surgery?” Individuals who indicated they received medication for ADHD were also excluded because this may affect their motivation for exercise.

### Identification of Previously Diagnosed Diabetes

Individuals were considered to have diabetes if they reported ever being told by a health care provider that they had diabetes, excluding gestational diabetes. We also removed individuals with an A1C ≥6.5% to account for undiagnosed diabetes.

### Identification of Pre-diabetes

Individuals participating in the NHANES undergo a physical examination that includes laboratory analysis of blood. We defined diagnosed pre-diabetes as any respondents who reported being told that they had pre-diabetes or borderline diabetes. The specific wording was, “Have you ever been told by a doctor or other health professional ever been told by a doctor or other health professional that you have any of the following: pre-diabetes, impaired fasting glucose, impaired glucose tolerance, borderline diabetes or that your blood sugar is higher than normal but not high enough to be called diabetes or sugar diabetes?” We defined undiagnosed pre-diabetes among individuals without previously diagnosed or undiagnosed diabetes or diagnosed pre-diabetes using the A1C range of 5.7–6.4% (39–46 mmol/mol), as specified by the ADA (2).

We excluded individuals with previously diagnosed diabetes or pre-diabetes from the computation of undiagnosed pre-diabetes because the glycemic status of those individuals may simply have represented diabetes control. This recoded variable was binary.

### Depressive Symptomatology

Depressive symptomatology was assessed using the Patient Health Questionniare-9 (PHQ-9) (14). The reliability and validity of the PHQ-9 has been assessed both in the general population and in clinical samples (15). For the purposes of this analysis, individuals with a PHQ-9 score of 10 or greater were classified as having high depressive symptomatology.

The focus for this variable was on the self-report of symptoms for depression. There were some individuals who reported taking medication for depression. It was felt that with diagnosed depression whose depression was under control should not be classed as depression in relation to the likelihood of adherence with lifestyle change recommendations.

### Physician Recommended Lifestyle Modifications and Adherence

Patients who responded “Yes” to the interview question, “During the past 12 months have you ever been told by a doctor or health professional to control your weight or lose weight?” were classified as having received a recommendation from their physician to lose weight. Patients who further indicated that they were currently attempting to lose or control their weight were categorized as attempting to complete a recommended weight-loss lifestyle modification. The same criteria were used to establish attempting to complete a recommendation to exercise more based on their responses to the interview question, “During the past 12 months have you ever been told by a doctor or health professional to increase your physical activity or exercise?” and further indicated that they were currently trying to increase their physical activity or exercise habits.

### Demographics and Covariates

Age, sex and race/ethnicity was derived from the NHANES interview. Race/ethnicity was categorized into four groups: (1) Non-Hispanic White, (2) Non-Hispanic Black, (3) Hispanics and (4) Other. We also included whether individuals had seen a physician or other healthcare provider at least once in the past 12 months. We also assessed among who had been seen in an outpatient setting whether individuals had seen a mental health provider in the past 12 months.

### Statistical Analysis

All analyses were conducted using the survey package in R. v4.0.3. Weighting and design variables applied to all analyses to account for the stratified multistage probability sample design. Incorporation of the weighting and design variables allows us to calculate population estimates for the non-institutionalized US population. Unadjusted odds ratio from logistic regression models were used to determine the associations between pre-diabetes and follow-through on lifestyle modifications. Interaction terms between high depressive symptomatology and lifestyle modifications were used to test for the presence of effect modification from depressive symptoms. For analyses on weight-loss, individuals who had never received a recommendation to lose weight were excluded. Similarly for exercise, individuals who had never received a recommendation to exercise more were excluded from analyses. Additional logistic regression analyses exploring the adjusted relationship between attempting lifestyle modifications and high depressive symptomatology among patients with diagnosed pre-diabetes were also performed, adjusting for age, obesity, sex, outpatient health care in the past 12 months, and race/ethnicity.

## Results

The characteristics of the sample are shown in Table 1. The prevalence of individuals with pre-diabetes who also have high depressive symptomatology is not uncommon at 14.8%. This accounts for 6,736,053 adults in the US.

**Table 1:** Descriptive statistics of overweight or obese adults who have never been diagnosed with diabetes.

	**Overall**	**Undiagnosed pre-diabetes**	**Diagnosed pre-diabetes**	**No pre-diabetes**
Unweighted sample size	3,453	761	448	2,244
Weighted sample size	141,851,135	28,790,161	18,992,903	94,068,071
**Age**				
18–39	38.8%	23.5%	23.8%	47.0%
40–70	50.6%	60.4%	60.9%	45.4%
71+	10.6%	16.1%	15.3%	7.5%
Gender (female)	48.0%	46.5%	50.7%	47.6%
**Race/ethnicity**				
Non-hispanic white	59.7%	55.6%	58.9%	61.3%
Non-hispanic black	12.1%	17.5%	9.7%	10.7%
Hispanic	18.7%	16.2%	22.7%	18.7%
BMI ≥30–34.9	30.1%	30.4%	29.2%	30.1%
BMI ≥35–39.9	14.3%	16.3%	18.5%	12.5%
BMI ≥40	10.9%	12.8%	13.5%	8.9%
High depressive symptomatology	7.2%	5.2%	6.7%	7.9%
Outpatient visit (past 12 mos.)	81.4%	83.0%	91.6%	79.6%
Mental health (past 12 mos.)	10.1%	6.8%	13.7%	10.7%
Told to lose weight	30.4%	29.5%	54.9%	24.3%
Trying to lose weight	24.6%	23.0%	43.6%	20.0%
Told to exercise more	40.5%	40.2%	67.4%	33.9%
Trying to exercise more	29.0%	25.9%	48.4%	25.3%

Table 2 shows the unadjusted odds ratios between lifestyle modifications and pre-diabetes stratified by depression. Individuals diagnosed with pre-diabetes had lower odds of attempting physician-recommended weight-loss and exercise-oriented lifestyle modifications than individuals without diagnosed pre-diabetes among patients with depression. However, no statistically significant associations between attempting lifestyle modifications and diagnosed pre-diabetes were observed among patients with low depressive symptomatology. A statistically significant interaction term between depressive symptomatology and exercise suggests that depressive symptomatology on may be an effect modifier for attempting recommended changes in physical activity among patients with diagnosed pre-diabetes, though the same effect was not seen for weight loss recommendations. There were no statistically significant interactions between depressive symptomatology and lifestyle modifications for patients with undiagnosed pre-diabetes.

**Table 2:** Unadjusted odds ratios and 95% confidence intervals for the associations between pre-diabetes and trying to complete lifestyle changes after a doctor's recommendation stratified by depressive symptomatology.

	**High depressive symptomatology**	**Low depressive symptomatology**	***P* ^**‡**^**	**High depressive symptomatology**	**Low depressive symptomatology**	***P* ^‡^**
	**Undiagnosed pre-diabetes vs**.			**Diagnosed pre-diabetes vs**.		
	**diagnosed/no pre-diabetes**			**undiagnosed/no pre-diabetes**		
Trying to lose weight	1.24 (0.42, 3.65)	0.75 (0.40, 1.39)	0.462	0.28 (0.10, 0.76)	0.97 (0.46, 2.05)	0.102
Trying to exercise more	2.00 (0.55, 7.30)	0.60 (0.38, 0.95)	0.162	0.24 (0.08, 0.75)	1.16 (0.68, 1.98)	0.027

‡ *P-values represent the significance of an interaction between major depression and trying to complete a lifestyle recommendation*.

Additional adjusted analyses were used to confirm the associations between diagnosed pre-diabetes and attempting to complete a lifestyle intervention. Among patients with diagnosed pre-diabetes, depressed patients were less likely to attempt to exercise more (OR = 0.31; 95% CI: 0.10, 0.94) and no association between high depressive symptomatology and attempting to lose weight was observed (OR = 0.45; 95% CI: 0.14, 1.42).

## Discussion

The findings of this study indicate that many patients with pre-diabetes also have high depressive symptomatology. Depressed, overweight patients with diagnosed pre-diabetes are less likely to follow physician recommendations for exercise in a lifestyle modification. However, the same is not true of overweight patients with low depressive symptomatology, whose attempts to follow physician-recommended lifestyle modification to treat diagnosed pre-diabetes is not hindered by depressive symptoms. Lack of motivation and cognitive impairments are the main symptoms preventing depressed patients from attempting lifestyle modification. A core symptom of depression is anhedonia, defined as a “markedly diminished interest or pleasure in all, or almost all, activities most of the day, nearly every day” (16). Anhedonia leads to reductions in physical and cognitive effort, both components in goal-directed, motivated behavior (17). Motivation is interdependent with cognitive control, which is also deficient in depressed patients and leads to impairments in attention, interpretation, and memory. Taken together, deficits in motivation and cognition make it challenging for patients with depression to accomplish tasks and goals.

Depression affects a patient's ability to attempt exercise compared to the ability to lose weight, which is not surprising. The daily fatigue, psychomotor retardation (being slowed down), and feelings of worthlessness often experienced by depressed patients make it difficult to exercise, while the loss of appetite and unintentional weight loss typical of depression may have made it appear that intentional weight loss was easier (16).

The results of this study are consistent with some past research which showed that adherence to an intense lifestyle intervention is lower for patients with depression (18). Previous research has shown the effectiveness of pharmacotherapy (i.e., metformin) for diabetes prevention (19). Although the National Diabetes Prevention Program (National DPP) showed that both an intense lifestyle intervention and metformin were effective strategies for diabetes prevention, the lifestyle intervention had greater benefits. However, the DPP did not consider depression or depressive symptomatology, and it may be that the effectiveness of an intense lifestyle intervention in actual practice may have significantly lower adherence than metformin among patients who are depressed. This research suggests that an alternative to a lifestyle intervention may be warranted. Future research should focus on this considering the millions of patients with pre-diabetes and depression.

There are several limitations to this study which need to be noted. First, the information collected in the NHANES on physician recommendation for lifestyle change and reports of attempts for lifestyle change are based on self-reports. Thus, the lifestyle interventions may vary and may not be as intense as the National DPP. However, the current results suggest that individuals with high depressive symptomatology are still less likely to do exercise programs regardless of the range of intensity suggested by the physicians. Moreover, the NHANES is a survey and is not a chart audit and so aspects of the respondent's medical history, if not collected in the survey with specific questions, will not be available for analysis. Second, the patients reported their depressive symptomatology. This is not a diagnosis via a clinical interview. It should be noted that as was expected, the effect was found based on whether individuals exhibit the depressive symptomatology that may impact on adherence to lifestyle changes. Third, although the results are nationally representative based on population weighting and the adjusted analyses control for race/ethnicity, a subpopulation only analysis could yield different results.

In conclusion, this study indicates that millions of people with pre-diabetes have significant depressive symptomatology, and this may affect the effectiveness of prescribing an intense lifestyle intervention for diabetes prevention. The next step is to determine in a more controlled fashion whether lifestyle interventions or pharmacotherapy are equally effective for diabetes prevention among patients with depression.

## Data Availability Statement

The original contributions presented in the study are included in the article/supplementary material, further inquiries can be directed to the corresponding author/s.

## Author Contributions

AM led the analysis plan, wrote the first draft, and is the guarantor of the paper. BR conducted the analysis. All authors contributed to the initial idea, final writing, and editing of the manuscript.

## Conflict of Interest

The authors declare that the research was conducted in the absence of any commercial or financial relationships that could be construed as a potential conflict of interest.

## Publisher's Note

All claims expressed in this article are solely those of the authors and do not necessarily represent those of their affiliated organizations, or those of the publisher, the editors and the reviewers. Any product that may be evaluated in this article, or claim that may be made by its manufacturer, is not guaranteed or endorsed by the publisher.
